# Active Immunotherapy for the Prevention of Alzheimer’s and Parkinson’s Disease

**DOI:** 10.3390/vaccines12090973

**Published:** 2024-08-28

**Authors:** Madeline M. Vroom, Jean-Cosme Dodart

**Affiliations:** Vaxxinity, Inc., Space Life Sciences Lab, 505 Odyssey Way, Merritt Island, FL 32953, USA; madeline@vaxxinity.com

**Keywords:** vaccines, active immunotherapy, neurodegenerative disease, Alzheimer’s disease, Parkinson’s disease

## Abstract

Neurodegenerative diseases (ND) give rise to significant declines in motor, autonomic, behavioral, and cognitive functions. Of these conditions, Alzheimer’s disease (AD) and Parkinson’s disease (PD) are the most prevalent, impacting over 55 million people worldwide. Given the staggering financial toll on the global economy and their widespread manifestation, NDs represent a critical issue for healthcare systems worldwide. Current treatment options merely seek to provide symptomatic relief or slow the rate of functional decline and remain financially inaccessible to many patients. Indeed, no therapy has yet demonstrated the potential to halt the trajectory of NDs, let alone reverse them. It is now recognized that brain accumulation of pathological variants of AD- or PD-associated proteins (i.e., amyloid-β, Tau, α-synuclein) begins years to decades before the onset of clinical symptoms. Accordingly, there is an urgent need to pursue therapies that prevent the neurodegenerative processes associated with pathological protein aggregation long before a clinical diagnosis can be made. These therapies must be safe, convenient, and affordable to ensure broad coverage in at-risk populations. Based on the need to intervene long before clinical symptoms appear, in this review, we present a rationale for greater investment to support the development of active immunotherapy for the prevention of the two most common NDs based on their safety profile, ability to specifically target pathological proteins, as well as the significantly lower costs associated with manufacturing and distribution, which stands to expand accessibility to millions of people globally.

## 1. Introduction to Alzheimer’s and Parkinson’s Disease

Neurodegenerative disease—an umbrella term for conditions wherein the pathological deterioration of neurons yields clinical impairments in motor/cognitive capabilities—poses a significant challenge to society and public health on a global scale. Indeed, dementia affects an estimated 55 million people, is the fifth leading cause of death worldwide, and its economic burden is projected to increase from USD 2.8 trillion in 2019 to USD 16.9 trillion by 2050 [1,2,3]. The prevalence of NDs combined with their debilitating socioeconomic toll make mitigative and preventative therapies against Alzheimer’s disease (AD) and Parkinson’s disease (PD)—the two most prevalent neurodegenerative conditions—a critical, unmet need of modern-day medicine.

Alzheimer’s Disease is the most common ND, accounting for 75% of dementia-type diagnoses [4]. Anatomically, AD is demarcated by prominent atrophy in the hippocampus and cerebral cortex of the brain, which gives rise to cognitive dysfunction and progressive memory loss and erodes an individual’s ability to perform daily functions. The pathological hallmarks of AD include the formation of extracellular plaques and intracellular neurofibrillary tangles, which impede synaptic function and are, respectively, composed of amyloid-β (Aβ) and Tau proteins [4,5,6]. Parkinsonism is broadly defined as a class of movement disorders that present with bradykinesia (i.e., slowing of movement), rigidity, tremors, and postural instability [7]. Parkinson’s Disease, which accounts for 80% of Parkinsonism diagnoses, is second only to AD in terms of prevalence. Parkinson’s Disease is characterized by the loss of neurons in the central and peripheral nervous systems, causing pronounced atrophy of the midbrain (i.e., substantia nigra) accompanied by motor impairment and cognitive decline. On a neuronal level, PD is pathologically characterized by irregular inclusions of the pre-synaptic protein α-synuclein [7,8,9].

**Figure 1:**
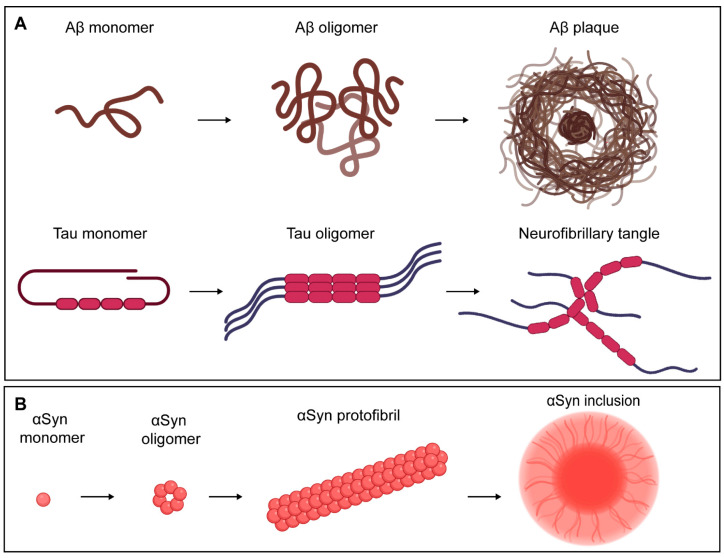
Aggregate-prone variants of endogenous proteins are associated with ND. (**A**) In Alzheimer’s Disease, pathological Aβ and hyperphosphorylated Tau proteins form amyloid plaques and fibrillary tangles, respectively. (**B**) In Parkinson’s Disease, αSyn oligomerizes into protofibrils that aggregate further to form intracellular inclusions. Image created with BioRender.

Although AD and PD are distinct diseases, both are neurodegenerative and possess several key similarities. First, although genetic etiology undoubtedly plays a role in certain populations, the principal risk factor associated with AD or PD is age [10]. This has amplified the problem posed by NDs to society, as global life expectancy has steadily increased over the last several decades. For example, between 1980 and 2019, the average life expectancy increased from 61.0 to 72.5 years [11]. Second, both AD and PD are characterized by toxic gain-of-function in endogenous proteins that serve physiological roles in a non-diseased state. Amyloid-β (Aβ) and Tau have been shown to support synaptic plasticity and neurogenesis, whereas α-synuclein (αSyn) is known to interact with neurotransmitter regulators at pre-synaptic termini [12,13,14,15]. Third, the toxicity of these proteins is largely potentiated by the enrichment of pathological variants with a high propensity for aggregation (Figure 1). AD plaques are enriched for Aβ_1–42_, which is produced from the differential cleavage of the amyloid precursor protein and is more prone to oligomerization than Aβ_1–40_, which normally accounts for 90% of total Aβ [16,17]. The hyperphosphorylation of Tau induces both its dissociation from microtubules as well as the formation of AD neurofibrillary tangles, and αSyn inclusions are well-known as a molecular aberration that underpins the pathogenesis of PD [18,19]. Fourth, these aggregate-prone toxic variants are detectable in the brain long before the disease becomes clinically apparent. Aβ and Tau accumulation has been shown to begin as much as 15 years prior to symptom onset, whereas increases in pathological αSyn are quantifiable in cerebrospinal fluid (CSF) up to 3 years before the early motor stage of PD [14,20].

## 2. The Need for a Preventative Treatment Paradigm in ND

Currently, there are no drugs capable of stopping, reversing, or preventing AD or PD, and most pharmacological agents (e.g., cholinesterase and N-methyl-D-aspartate inhibitors for AD, monoamine oxidase inhibitors and levodopa for PD) seek to alleviate symptoms rather than alter the progression of the disease [5,21,22]. However, monoclonal antibody (mAb) therapies against aggregated Aβ as passive immunotherapies have recently demonstrated disease-modifying potential and the capacity to reduce the rate of clinical decline [23]. Specifically, a post-hoc analysis of the Phase II results from the PASADENA study revealed that prasinezumab (anti-αSyn) slowed motor deterioration in rapidly progressing, early-stage PD [24]. Aducanumab, lecanemab, and donanemab (anti-Aβ) were granted FDA approval in 2021, 2023, and 2024, respectively, based on modest reductions in the decline of cognition, function, and behavior for patients with early AD [25,26,27]. Although hailed as a paradigm shift in ND treatments by some, the clinical outcomes achieved with these mAbs are modest, and whether they translate into meaningful, tangible benefits for patients remains a subject of debate among experts [28,29]. In fact, the Federal Drug Administration’s (FDA) 2021 approval of aducanumab—which lessened the rate of neurocognitive decline by 22% relative to the control in the Phase III EMERGE trial—was highly controversial due to the fact that the agency’s own Advisory Committee ruled against its release on the basis of lack of efficacy in the second Phase III ENGAGE trial, as well as treatment-related amyloid-related imaging abnormalities (ARIA), which indicate edema and cerebral microhemorrhages [30,31,32]. Lecanemab and donanemab, both of which are anti-amyloid mAbs for AD, possess similar safety and efficacy profiles [27,33].

Unfortunately, although passive immunotherapies have boasted transformative success in the treatment of high cholesterol, rheumatic disease, and certain cancers, the same cannot be said for AD or PD [34,35,36,37,38]. Currently, the best-case scenario for passive immunization is a modest, short-term, reduction in clinical decline that may offer several months or years of better-quality life [39]. However, even this non-curative endpoint has proven difficult to achieve, and the limited number of therapeutic mAbs for AD (Lecanemab, donanemab) and PD (none approved) is not for want of investment or effort. Indeed, many mAbs have demonstrated promising safety and binding profiles against pathological species of Aβ, Tau, and αSyn but failed to achieve efficacy in Phase II and III trials [40,41]. Notably, although lecanemab treatment significantly reduced or eliminated Aβ plaque burden in early AD patients after 18 months, the rate of clinical decline was lessened by a mere 27%, prompting the conclusion that “the modest reduction in rate of clinical decline may not be evident to most affected individuals” [42]. Similar results were obtained from the Phase III trials with aducanumab [40]. Evidently, the clearance of a pathological protein and its hallmark aggregate structures is insufficient to arrest ND progression or markedly improve clinical function, even during the early stages of the disease [43]. Considering the brain atrophy and protein aggregation that predates notable deficits in motor skills and cognition, the persistent failure of mAbs to substantially slow motor/cognitive decline during the early clinical stages of AD or PD strongly suggests brain pathology is too advanced at the time of clinical diagnosis for disease-modifying therapeutics to be highly impactful [44]. Although the therapeutic value of a complete blockade against pathological Aβ, Tau, or αSyn has yet to be fully elucidated, a compelling argument thus arises for the need to focus drug development efforts on the preclinical stage of AD or PD and preventive treatments that inhibit the accumulation and aggregation of toxic Aβ/Tau or αSyn species. Indeed, initiating therapy before the onset of clinical symptoms extends the timeline for pharmacological intervention in support of preserving neuronal viability, protecting motor/cognitive function, and, ideally, preventing ND in its entirety.

## 3. The Case for Vaccines as a Preventative against ND

The first vaccine was described in the 18th century when Edward Jenner discovered that inoculation with attenuated cowpox virus conferred immunity against smallpox [45]. The subsequent evolution of germ theory, advances in bacterial/viral cultivation techniques, and the introduction of recombinant DNA and sequencing technology gave rise to vaccines that have revolutionized humanity’s experience of infectious disease on a global scale [46]. This is, undoubtedly, best exemplified by the global eradication of smallpox in 1980 following a sustained immunization campaign [47]. Over the last several decades, vaccine research and development efforts have expanded beyond infectious disease to include potential preventive measures against chronic, non-communicable illnesses that derive from endogenous “self” antigens, including cancer, dyslipidemia, diabetes, and NDs [48]. Although they share a common premise, vaccines against endogenous targets are necessarily different from those for infectious diseases, and numerous regulatory agencies, including the FDA, limit the definition of vaccines to infectious disease indications [49]. Hence, “active immunization” and “active immunotherapy” will be used forthwith when referencing the pharmacological induction of antibodies against endogenous AD or PD targets.

Based on the need to intervene years before the onset of clinical ND symptoms, preventative therapies for AD and PD must meet several practical requirements as a matter of feasibility. The first criterion is ease of administration and cost. That is, to ensure broad coverage of the general population and democratized access, a prophylactic agent against ND must be both convenient to administer and affordable to obtain. The arduous dosing regimen of mAbs and their prohibitive price (e.g., an hour-long IV infusion every 2 weeks for 18 months and USD 26,500 annually for lecanemab [50]) make passive immunotherapy ill-suited for preventative purposes. The second criterion is specificity. Given that normal Aβ, Tau, and αSyn perform physiological functions and are not known to play a role in pathogenesis, it is critical for any drug to target the toxic gain-of-function variants associated with the disease state to avoid potential adverse side effects. Third, given that the risk of AD or PD increases with age, a preventative therapy against ND must be safe for use in middle-aged and geriatric populations [10]. With these criteria in mind, one type of therapeutic stands out as being particularly well-suited for use as a prophylactic agent against AD or PD—namely, active immunotherapies which stimulate the immune system to induce the production of protective, antigen-specific, antibodies [23,51].

Active immunization may provide several distinct advantages over other types of therapeutic modalities for NDs. Potential routes of administration include intramuscular (IM) injections, oral drops, and nasal sprays, and several ongoing Phase II clinical trials feature the use of microneedle patch devices [52,53,54,55]. Following the initial priming regimen, boost inoculations typically occur on the order of months, if not years, and have the potential to be self-administered, thus increasing the rate of compliance [56]. Furthermore, although the cost efficacy of active immunotherapies may vary between high- and low-income countries, vaccine prices have historically been negotiable, and in many cases, the requisite infrastructure for public distribution has been established through national immunization programs [57,58,59]. Active immunization elicits an immune response and the production of antigen-specific antibodies; accordingly, the immunogen construct can be engineered against epitopes of the pathological, aggregate-prone, variants of Aβ, Tau, or αSyn to avoid impeding normal physiology [60]. Separately, although data concerning efficacy, immunogenicity, and safety in the elderly are limited, studies have generally found immunizations (i.e., against influenza, pneumococcal pneumonia, shingles) to be safe, well-tolerated, and revealed no specific concerns associated with age [61]. Finally, active immunization makes it possible to target multiple AD or PD epitopes simultaneously via multivalent formulation, which raises the prospect of a single prophylactic against both Alzheimer’s and Parkinson’s Disease.

## 4. The Safety of Active Immunotherapies for ND Prevention

To date, there has been a disproportionate investment in passive immunotherapy (i.e., mAbs) for the treatment of AD or PD as opposed to active immunotherapy for prevention. Much of this reticence is the legacy of AN-1792, an anti-Aβ vaccine for AD that was abruptly terminated mere months into a highly anticipated phase II clinical trial in individuals with mild to moderate AD, due to multiple patients developing aseptic meningoencephalitis after immunization, which in some instances proved fatal [62]. AN-1792 was the first immunotherapy to deliberately induce a protective antibody response in humans against an endogenous target also found in healthy tissue and, in the aftermath of the trial, many experts rightly questioned whether active immunization could safely be used to treat or prevent NDs [63]. However, follow-up studies revealed that the neuroinflammation induced in approximately 6% of immunized patients was likely a byproduct of the immunogen and adjuvant. More specifically, AN-1792 contained synthetic, full-length, Aβ_1–42_ peptide, which possesses inherent T-cell epitopes [64]. Moreover, the test article was formulated with QS-21, a potent proinflammatory adjuvant, the effects of which were further amplified by polysorbate 80 [65,66]. Consequently, immunization induced a systemic, proinflammatory, Th1-biased immune response that, in some individuals, led to T-lymphocyte infiltration of the brain and meningoencephalitis [67,68]. The conspicuous absence of such adverse reactions in the Phase I trial for AN-1792 likely derives from test article instability, as the de-acylation of QS-21 yields a Th2-biased response and reduced adjuvanticity [66,69].

Certainly, the case of AN-1792 emphasizes the need for stringent quality control and safety testing in the development of active immunotherapies against endogenous “self” antigens. However, it would be erroneous to conclude that the therapeutic modality is ubiquitously unsafe for NDs. Although autoimmune in nature, active immunotherapy is not to be confused with autoimmune disease, wherein aberrant B and T cell responses against self-antigens lead to dysfunction and damage [70]. The antibody response induced by active immunotherapies can be engineered to specifically target pathological Aβ, Tau, and αSyn and could facilitate the natural antibody response observed in patients with AD or PD against those pathological targets, especially since naturally occurring autoantibodies do not seem to be produced at therapeutic concentrations [71,72]. Leveraging the lessons learned from AN-1792, numerous active immunotherapies against Aβ, Tau, and αSyn have been developed over the last two decades for AD or PD, some of which have entered clinical testing [43,73,74,75,76,77]. A summary of these active immunotherapies and their most recent clinical findings is presented in Table 1. Importantly, none of these second-generation therapeutics have been found to induce meningoencephalitis and were generally safe and well-tolerated in clinical trials. Based on the data available, the incidence of amyloid-related imaging abnormalities (ARIA) is much lower with active immunization than is typically observed with mAbs [78]. In fact, the main side effect observed with AD and PD active immunotherapies is a localized injection site reaction that typically resolves without intervention within a matter of days. Thus, the safety profile of modern active immunotherapies against AD and PD compares favorably with active immunotherapies against infectious diseases.

## 5. Current Challenges for AD and PD Active Immunotherapies

Although active immunization possesses clear strategic advantages as a prophylactic for AD or PD, there are numerous obstacles that must be surmounted on the road to success. In particular, although most active immunotherapies have demonstrated encouraging effects in preclinical models of disease, these effects have not yet translated into clinical efficacy. This could be attributed, in part, to the suitability of animal models that need to be predictive of both the human immune response and the human disease. Current investigational active immunotherapies might be targeting the right pathological peptides or proteins; however, it remains to be demonstrated that immunization raises antibody levels sufficiently high to neutralize and/or clear the toxic species. As a matter of fact, the pharmacology of active immunotherapies that preferentially stimulate a humoral response has been poorly studied and further investigation is necessary to understand the optimal doses, the dose regimens, and the actual antibody levels, as well as the quality of the antibodies produced in humans. Finally, prevention clinical trials come with their own challenges; they would necessarily be lengthy and involve very large cohorts of individuals. Enriching cohorts with individuals developing the disease but still asymptomatic requires costly and constraining brain imaging or cerebrospinal fluid collection. Currently, intense research efforts are ongoing to validate convenient blood-based biomarkers of disease that would facilitate the identification of individuals with preclinical AD or PD, i.e., individuals with brain pathology who have not yet shown clinical signs of disease.

Active immunotherapies currently in the clinic have demonstrated acceptable safety and tolerability profiles so far, certainly owing to better epitopes and adjuvants selection. To avoid adverse events such as those caused by AN-1792, active immunotherapies for NDs have largely focused antigen selection efforts on peptide sequences from toxic gain-of-function variants of Aβ, Tau, and αSyn that do not possess intrinsic T-cell epitopes. In lieu of these proinflammatory motifs, the peptide fragments are strategically replaced with T helper peptide carriers and adjuvants intended to induce a potent but safe humoral immune response against the ND epitope of choice. The carriers and delivery systems of those treatments that have entered clinical testing include naturally occurring and synthetic peptide immunogens (i.e., *Clostridium tetani* and *Diphtheria pertussis* toxin derivatives, UBITh), proteins (i.e., keyhole limpet hemocyanin), virus-like particles, and liposomes, whereas the corresponding adjuvants have included QS-21, alum with or without CpG oligonucleotide, the oil-in-water nano-emulsion MF59, and monophosphoryl-lipid A [68,94,95,96,97,98]. Although several active immunotherapies for AD or PD are the subject of ongoing Phase II trials, a more systematic approach to the formulation and detailed understanding of the resultant immune response, which is still in its nascency, may be required to achieve a meaningful impact in the clinic. Thus far, however, efficacy has not yet been demonstrated in the clinic. Most trials have involved relatively small patient populations (i.e., in the order of hundreds) and have only investigated one or two dose regimens. Thus, additional dose-finding studies would be necessary to fully understand how to maximize the antibody response, achieve high responder rates, and to address whether meaningful antibody concentrations can be achieved to engage the pathological target. Moreover, data focused on the characterization of antibodies produced in patients are very sparse. Most of the antibody characterizations have only focused on the specificity of the antibodies towards various toxic species over the physiological peptides or proteins [78,87,89,93,99].

The understanding that AD or PD brain pathology manifests years before the onset of clinical symptoms represents a relatively recent paradigm shift in ND. Indeed, the concept of a preclinical stage for AD or PD was introduced in the early 2000s and it is only in the last two decades that the significance of this asymptomatic phase has truly become apparent [100,101,102,103]. As a result, emerging drugs to prevent, slow, or arrest the progression of AD or PD have increasingly refocused development efforts on the preclinical stage of the disease, which offers a better opportunity to tackle the neuropathological processes prior to dramatic neurodegeneration. However, in lieu of the physical and neurological exams used to traditionally diagnose and stage symptomatic AD or PD, prevention studies require the identification of suitable populations for enrollment, as well as well-defined and relevant endpoints [44,104]. Large-scale trials (i.e., in the order of tens of thousands) are further complicated by the heavy financial burden associated with running comprehensive, longitudinal, prevention studies, and the regulatory path is not yet well established. Currently, the best practices for identifying individuals in the preclinical stage of AD or PD include various imaging modalities, such as structural/functional magnetic resonance imaging (MRI) and positron emission tomography (PET), as well as CSF biomarkers [101,105,106,107,108]. Unfortunately, these approaches lack scalability due to expense, lack of accessibility, and/or physical invasiveness [44,109]. However, there have been numerous advancements towards determining suitable blood-based biomarkers for the identification of individuals presenting with preclinical AD (i.e., plasma Aβ_1–42_/Aβ_1–40_ ratio and Tau phosphorylated at residues 181, 217, or 231). Similarly, potential biomarkers of PD pathology are being investigated in various peripheral tissues such as blood or skin (pathological αSyn, neurofilament light chain, glial fibrillary acidic protein). Further validation of those biomarkers is ongoing across multiple institutions and will certainly facilitate the design and execution of prevention studies for AD or PD on the scale necessary to properly assess the prophylactic potential of active immunotherapies [110,111,112,113,114,115].

## 6. Conclusions

In conclusion, mounting evidence indicates that therapeutic intervention against AD or PD must occur years before the manifestation of clinical symptoms to alter the trajectory of the disease in a meaningful way. In considering those pharmacological agents with the potential to be repurposed as prophylactic agents against NDs, active immunotherapies are particularly well-suited based on their ease of administration, financial accessibility, and the capacity to target pathological Aβ/Tau/αSyn in a safe and highly specific manner. The present-day challenges regarding epitope selection, formulation, identification of suitable translational models, and the need for scalable diagnostic metrics to support high-powered primary prevention trials are substantial but surmountable, and warrant further investment in active-type immunotherapies against Aβ/Tau and αSyn. Indeed, such drugs comprise one of modern medicine’s most promising strategies to significantly reduce the worldwide incidence of AD and PD. Furthermore, active immunotherapy may be applied to other NDs (e.g., frontotemporal dementia) once suitable targets are identified, and in so doing, stands to transform how humankind experiences chronic disease in the 21st century.

## Figures and Tables

**Table 1:** Summary of AD or PD active immunotherapies that have completed clinical trials and their most recent findings.

Target/Therapy	Status	Epitope	Phase	Participants	Findings
Aβ/CAD106	Discontinued	Aβ(1–6)	II, III	Cognitively unimpaired APOE ε4 homozygotes, 60 to 75 years of age	Therapy was safe and well tolerated. Modest anti-Aβ titers induced, slower Aβ plaque formation [79].
Aβ/ACC-001	Discontinued	Aβ(1–7)	II	Individuals 50 to 85 years old with mild to moderate AD	Therapy was safe and well tolerated. Anti-Aβ titers induced in all groups [80]
Aβ/Lu AF20513	Discontinued	Aβ(1–12)	I	Individuals 60 to 85 years old with diagnosis of probable AD.	Results not posted; studies were terminated due to efficacy data from a new study [81,82,83]
Aβ/UB-311	Active	Aβ(1–14)	II	Individuals between 60 and 90 and a diagnosis of mild AD	Therapy was safe and well tolerated with a 97% antibody response rate [78,84]
Aβ/ACI-24	Active	Aβ(1–15)	II	Individuals between 25 and 45 years of age with Down Syndrome and a genetic risk of developing AD	Therapy was safe and well tolerated. Titer responder rate was only 33%. Increased Aβ_1–42_ observed in immunized groups [85]
Aβ/V950	Discontinued	Aβ(1–15)	I	Individuals 55 years and older with mild to moderate AD.	High rate of serious adverse effects (most commonly arrythmia, cerebrovascular accident). Few treatment groups exhibited titers 2-fold higher than placebo after 7 months [86]
Aβ/ABvac40	Active	Aβ(33–40)	II	Individuals 55 to 80 years old with mild cognitive impairment or very mild AD	Therapy was safe and well tolerated. No related PET abnormalities observed. Antibody response was strong and specific [87]
Aβ/AFFITOPE AD01	Discontinued	Aβ N-terminus mimetic	I	Individuals 50 years or older with diagnosis of probable mild/moderate AD	Detailed results not posted; therapy was found to be safe and well tolerated [88]
Aβ/AFFITOPE AD02	Discontinued	Aβ N-terminus mimetic	II	Individuals 50 to 80 years old with early AD	Therapy was safe and well-tolerated, but no significant treatment effects were observed [88,89]
Aβ/AFFITOPE AD03	Discontinued	Aβ N-terminus mimetic	I	Individuals 50 to 80 years, diagnosis of likely mild or moderate AD	Detailed results not posted; therapy was found to be safe and well-tolerated [88]
Tau/AADvac1	Active	Tau (294–305)	II	Individuals 50 to 85 years old with diagnosis of probable mild AD	Therapy was safe, well tolerated, and elicited robust IgG titers. No significant effects were observed in cognitive and functional tests [90]
Tau/ACI-35	Active	Tau (393–408)	II	Individuals 50 to 75 years old with mild cognitive impairment due to AD or mild AD	Therapy was safe, well tolerated, with a 100% response after the first injection. IgG preferentially targets phospho-Tau. IgM response was also induced [91]
α-Syn/AFFITOPE PD01A	Active	α-syn C-terminus mimetic	I	Individuals 40 to 65 years old with early PD	Therapy was safe and well tolerated. Immunogenicity was induced against the peptide and the target epitope over time [92]
α-Syn/AFFITOPE PD03A	Active	α-syn C-terminus mimetic	I	Individuals 45 to 70 years old with early PD	Therapy was safe and well tolerated. Immunogenicity was induced against the peptide and the target epitope over time [92]
α-Syn/UB-312	Active	α-syn C-terminus mimetic	I	Individuals 40 to 85 years; healthy or with PD diagnosis	Therapy was safe and well tolerated. Antibody responder rate was dose-dependent. Titers increased during the priming regimen and naturally decreased in the absence of additional boosts [93]
